# The emergence of complexity and restricted pleiotropy in adapting networks

**DOI:** 10.1186/1471-2148-11-326

**Published:** 2011-11-07

**Authors:** Hervé Le Nagard, Lin Chao, Olivier Tenaillon

**Affiliations:** 1 INSERM, UMR-S 722, Paris, F-75018, France; 2 INSERM, UMR-S 738, Paris, F-75018, France; 3 Univ Paris Diderot, Sorbonne Paris Cité, UMR-S 722 INSERM, F-75018, Paris, France; 4 Univ Paris Diderot, Sorbonne Paris Cité, UMR-S 738 INSERM, F-75018, Paris, France; 5 Institut Claude Bernard, IFR2, F-75018, Paris, France; 6 Division of Biology, University of California San Diego, La Jolla, California, USA

## Abstract

**Background:**

The emergence of organismal complexity has been a difficult subject for researchers because it is not readily amenable to investigation by experimental approaches. Complexity has a myriad of untested definitions and our understanding of its evolution comes primarily from static snapshots gleaned from organisms ranked on an intuitive scale. Fisher's geometric model of adaptation, which defines complexity as the number of phenotypes an organism exposes to natural selection, provides a theoretical framework to study complexity. Yet investigations of this model reveal phenotypic complexity as costly and therefore unlikely to emerge.

**Results:**

We have developed a computational approach to study the emergence of complexity by subjecting neural networks to adaptive evolution in environments exacting different levels of demands. We monitored complexity by a variety of metrics. Top down metrics derived from Fisher's geometric model correlated better with the environmental demands than bottom up ones such as network size. Phenotypic complexity was found to increase towards an environment-dependent level through the emergence of restricted pleiotropy. Such pleiotropy, which confined the action of mutations to only a subset of traits, better tuned phenotypes in challenging environments. However, restricted pleiotropy also came at a cost in the form of a higher genetic load, as it required the maintenance by natural selection of more independent traits. Consequently, networks of different sizes converged in complexity when facing similar environment.

**Conclusions:**

Phenotypic complexity evolved as a function of the demands of the selective pressures, rather than the physical properties of the network architecture, such as functional size. Our results show that complexity may be more predictable, and understandable, if analyzed from the perspective of the integrated task the organism performs, rather than the physical architecture used to accomplish such tasks. Thus, top down metrics emphasizing selection may be better for describing biological complexity than bottom up ones representing size and other physical attributes.

## Background

The evolution of the complexity of organisms has been a challenge for Darwinian theories of evolution [1]. How does evolution produce complex organs, when the functioning of such organs requires the successful interaction of many components? Despite the recent proliferation of large nucleotide, proteomic, and metabolic databases, it remains difficult to define the complexity of organisms [2,3], and even more to understand the determinants underlying the emergence of complexity.

Any attempt to understand the evolution of complexity must rely on a meaningful definition of complexity coupled to some quantitative methods of estimation. Initial estimates of complexity have been based on the number of nucleotides, genes or cell types in a genome, but such bottom up estimates often fail to have useful properties [4,5]. For instance, the multicellular green algae *Volvox carteri *has the same number of genes as its unicellular relative, *Chlamydomonas reinhardtii *[6], even though the evolution of multicellularity is one of the major transitions affecting organismal complexity. To overcome possible problems associated with the previously mentioned bottom up metrics of complexity, recent studies have shifted to a more top down approach by incorporating population genetics [7], quantitative genetics [8] and ecology [9] and quantifying complexity from the perspective of natural selection acting on the organism as a whole [10-12].

The most integrated vision of complexity comes from Fisher's geometric model of adaptation [13]. Fitness in the model is a function of the phenotype of the organism. Individual organisms are depicted as points in a multidimensional space and each axis corresponds to an independent phenotype under selection. The total number of independent phenotypes, i.e. the dimensionality of the phenotypic space, is taken to represent phenotypic complexity. This model has received much attention in the last decade and has provided many qualitative predictions [7,11,12,14-18] that have been validated experimentally [12,19,20].

Using Fisher geometric model of adaptation, several theoretical studies have also analyzed the consequences of phenotypic complexity on evolution. All of them found higher complexity to be costly. The cost results from the difficulty of having to optimize many phenotypes simultaneously and it is manifested by the decreasing fraction of beneficial mutations as dimensionality increases [11,13,15,21]. As a result, the rate of adaptation decreases [7,11,21,22] and the drift load increases [12,16,20,23-25]. Drift load represents the loss of fitness due the effects of genetic drift on the fixation rate of beneficial and deleterious mutations.

Although previous studies have used Fisher's geometrical model to examine the effect of complexity on evolution, none have allowed dimensionality to change as a result of evolution and adaptation. To characterize both the selective forces acting on the emergence of complexity and the underlying mechanisms, we have designed an evolutionary system in which complexity was free to emerge depending on its costs and benefits. Although experimental studies of complexity with real biological organisms are possible [11,12], a systematic investigation is still difficult. We chose therefore to use computational models employing artificial neural networks evolving asexually under a mutation-selection-drift process as an alternative.

## Methods

### Models

Neural networks were chosen over other models [26,27] because they offered more quantitative control over environmental challenges and network size, which is comparable to genome size. Neural networks can be seen biologically as a transduction signal pathway with no retroaction. In other words, depending on an initial input value, analogous to a chemical concentration, nodes are activated and provide a quantitative output equivalent to the transcription level of a gene. As such node-outputs/gene-expressions may affect the regulation of nodes/gene downstream in the network, the response to the initial chemical concentration is propagated through the network to the final node/gene. This node/gene is assumed to be directly linked to fitness such that the quantitative value of its output/expression can compared to some expected value to estimate fitness. In other words, for a given concentration of a chemical, a given expression level of the final gene is expected and any deviation from that value will reduce the fitness of the network. To go further, rather than evaluating the fitness of a network base only on its response to a single input value or concentration, we assess it on a linear gradient of concentrations. For 100 different concentrations spread between 0 and 1, we expect 100 different levels of expression of the output node. This means that fitness is defined as a goodness of fit between the output of the networks and a reference function. By allowing selection to operate through the fitness of the individual networks, the population was allowed to evolve.

**Figure 1:**
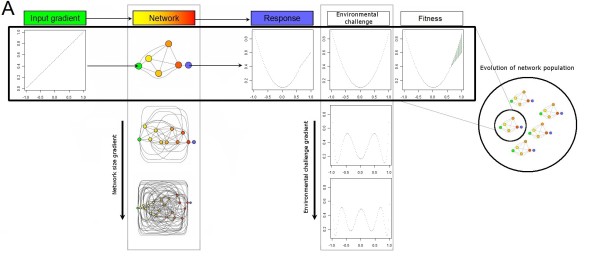
**Model of neural networks and environmental challenge**. All networks were evolved under an asexual mutation-selection-drift process. Fitness of an individual network was obtained by providing an input gradient and retrieving from the network a response that was then matched to a response function or environmental challenge. Networks varied in their number of nodes and connections and the weight of each connection and nodes could be mutated to generate heritable variation. Response functions were described by Legendre Polynomials. A higher Order of the Legendre Polynomial reflected a more intense environmental challenge. Populations of networks with identical size were adapted independently to each of the environmental challenges.

We used as the reference function Legendre polynomials. These functions were chosen because they could be readily ranked in term of complexity by the Order of the Legendre Polynomial (OLP). Higher OLP's both require more parameters and have a higher Kolmogorov complexity [28] (it requires a longer source code to be implemented, the size of the code increasing linearly with the OLP). Biologically, this means that a high OLP environment will select for networks whose response to a linear gradient of concentration is complex. For instance, if the expression of the key gene determines the state of the cell depending on a threshold level, as observed during development, an OLP of *p *selects for networks performing *p *transitions from one state to another along the full gradient. We note that while the reference function, and hence the environmental challenge could also be described as having either high or low complexity, we have chosen to restrict our use of the term complexity to describe only networks. Environmental challenges will be described as having different levels of demands: the higher the OLP, the more difficult is the environmental challenge (Figure 1)

**Figure 2:**
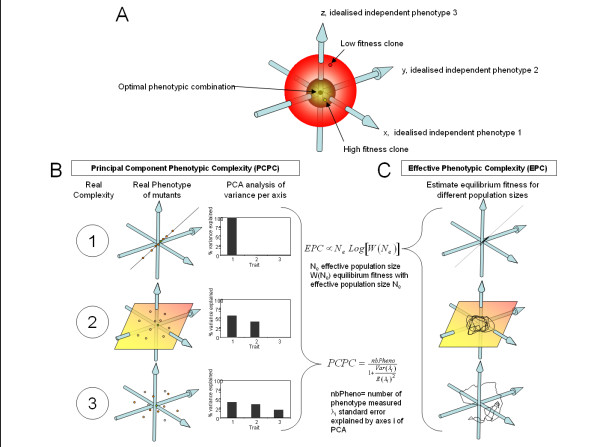
**A geometrical model of adaptation and the derived estimates of complexity**. A) A geometric model of adaptation is used as a reference to characterize complexity. In this model, an organism is defined by a number of idealized independent phenotypes (here 3). The number of phenotypes is what we will call phenotypic complexity. The model assumes the existence of an optimal combination of phenotype having maximal fitness. The more organisms are distant from that optimal combination, the lower is their fitness. B) To estimate phenotypic complexity, one can analyze a set of fitness-linked-phenotypes in a collection of mutants and perform a principal component analysis (PCA). The distribution of variance explained by the different axes of the PCA is directly linked to phenotypic complexity. For instance, if there is indeed a single phenotype (case 1), a single axis will explain all variance, while if complexity is indeed 2 or 3, 2 or 3 axis will be necessary to explain the phenotypic variance of mutants. C) Mathematical derivation from the geometric model have proved the existence of some fitness equilibrium and that the fitness at these equilibrium is a direct function of the effective population size and the phenotypic complexity. Hence if we record the average fitness of populations of different population size at equilibrium, we can estimate phenotypic complexity.

The evolution of complexity in our networks was monitored and compared to standard bottom up metrics such as network size with three additional metrics: Information Complexity (IC), Principal Component Phenotypic Complexity (PCPC), and Effective Phenotypic Complexity (EPC) (see detailed methods). IC measures the information content of the environment that is stored in a network by selection [10]. IC is based on a summation of the intensity of the selective constraints acting on each mutable component of the network. As such, IC estimation uses both bottom up (network size) and top down (selection) factors, it represents the functional size of the network and is a hybrid metric. Both PCPC and EPC are based on Fisher's geometric model of adaptation [13] (Figure 2A), which represents complexity as the dimensionality of the phenotypic space (the number of independent phenotypes under selection). PCPC directly measures Fisher's dimensionality from a principal component analysis based on the effects of randomly drawn mutation on the network fitness (Figure 2B) and is comparable to the dimensionality of the mutational variance-covariance M-matrix used in multivariate quantitative genetics [8]. EPC indirectly measures Fisher's dimensionality from the drift load [12] (Figure 2C). Drift load results from the impact of genetic drift on the fixation of deleterious and beneficial mutations. As the efficiency of selection depends on population size and the ratio of beneficial to deleterious mutations depends on dimensionality, dimensionality can be inferred from the intensity of drift load for different population size. Because EPC is easier to measure, it is one of the few indexes that have been measured in a real biological organism [12]. Because both PCPC and EPC respond only to selection, they are top down metrics.

## Detailed methods

### Networks

Neural networks consisted of single input and output cells connected by a series of neurons, or nodes (Figure 1). Network size was determined by varying the number of nodes. The input cell and nodes were connected sequentially and all nodes received out outputs from all lower nodes. The value of the output corresponds to the output of the last node. The output *O_j _*of node *j *was determined as

(1) $$Oj=1-e^{-x^{2}}$$

(different activation function provided similar results but with much lower efficiency of adaptation (data not shown)), where

(2) $$x=\left(\overset{j-1}{\underset{i=0}{\sum }}w_{ij}O_{i}+b_{j}\right)$$

*O_i _*were the outputs of the preceding nodes in the network, *w_ij _*the weight of the connection between node i and j (or connection weights), *b_j _*a weight associated to node j (or node weight), and *O_0 _*the input cell value. *O_j _*equaled zero when its inputs were zero and close to one with strong positive or negative inputs.

For each input value, the network provides an output value that can be compared to a reference. Rather than using the response to a single input value to define the fitness of the network, we used the response to 100 different input values. Fitness was hence determined by testing the response of each network to a gradient of values *g(i) *that increased linearly from -1 to +1 and *i *= 1 to 100 (g(i) = -1+2 × i/100). For each value *g(i)*, the network was evaluated by letting *O_0 _= g(i) *and measuring the response *N(g(i)) *in the output cell. Fitness was measured over all values of *i *as

(3) $$Fitness=e^{-K*{\left(\overset{100}{\underset{i=1}{\sum }}N\left(g\left(i\right)\right)-R\left(g\left(i\right)\right)\right)}^{2}∕\;\left(100*Var\left(F\right)\right)}$$

where *K *was a constant set to 5 and *R(g(i)) *the target value that *N(g(i)) *is supposed to match. The division by the variance of R(g(i)), *Var(F,) *normalized fitness to have comparable values for different forms of *R*.

Function *R *was a Legendre Polynomials (Figure 1). These are polynomials that define an orthogonal base for continuous functions defined between -1 and 1. In that interval, any function can be decomposed in a linear combination of Legendre polynomial of various orders. These polynomials are defined by the recursive functions

(4) $$\begin{array}{c} \left(n+1\right)P_{n+1}\left(x\right)=\left(2n+1\right)\;x\;P_{n}\left(x\right)-n\;P_{n-1}\left(x\right)\\ P_{0}\left(x\right)=1\\ P_{1}\left(x\right)=x \end{array}$$

For our purposes, we normalized them to be bounded by 0.1 and 0.9 in interval [-1,1].

Legendre polynomials of high order require many parameters to be defined and as such have a higher complexity than simple ones (in terms of Kolmogorov complexity [28], they require more operation to be encoded by a computer program). We therefore chose the Order of the Legendre Polynomial to reflect the demand of the network environment.

### Network evolution

Populations of networks were initially monomorphic, starting with a network having random weights (sampled in a uniform distribution between -0.5 and 0.5). The population of 500 networks was then submitted to a model of asexual evolution with discrete generations. Each generation, a network had a 1% probability of mutating one of its weights. The quantitative value of the mutated weight was then shifted by a random normal deviate of mean 0 and standard error 0.1. Using classical Wright-Fisher population genetics formalism, networks contributed to the next generation of networks according to their respective fitness.

The above evolutionary process represents our primary process of adaptation, however two other slightly modified evolutionary processes were used. We call the first modified process the "intense selection" evolutionary process. For that process, a smaller population size was used (50), but every 100 000 generations, the intensity of selection was increased, by increasing constant K, to set fitness back to 3%. This promoted an intense selective pressure that allowed the emergence of very high fitness clones that would have otherwise required very high population size (and massive amount of computer time) to emerge, as mutation of effects smaller than the inverse of population size behave as neutral mutations. Using this protocol fitness as high as 0.99999 were sometimes reached, while a population size of about 10^5 ^would be required for this level of fitness to be reached. The final evolutionary process we used can be called "adaptive dynamics" [29] since it is similar to the one used in this field. For that process, populations were always monomorphic except when a single mutant appeared. Then based on the mutant fitness *f_i _*relative to the resident fitness *f*_0_, the mutant either immediately invaded the population and became the new resident or disappeared. The probability of invasion P(*f*_0_→*f_i_*), depended on the evolving population size N and, using Sella and Hirsh formalism [30], was computed as:

(5) $$P\left(f_{0}\to f_{i}\right)=\frac{1-{\left(\frac{f_{0}}{f_{i}}\right)}^{2}}{1-{\left(\frac{f_{0}}{f_{1}}\right)}^{2N}}$$

This protocol provided an exact solution for populations having a small mutation rate by population size product. This evolutionary process allowed a faster computing than the process simulating a whole population; it limited the effects of high mutation rate by population size product that may lead to confusing effects and provided a direct access to the whole line of descent of the final clones. It allowed to follow of the coupled changes in fitness and complexity through the adaptive walk.

Similar levels of complexity were reached under all of these evolutionary algorithms. Our results are therefore robust and are not resulting from some specific selection favoring genetic robustness due to a high mutation supply.

**Figure 3:**
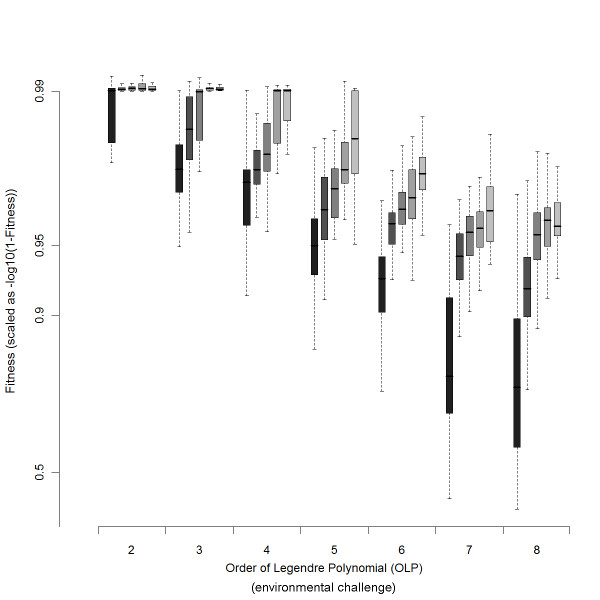
**Fitness reached by networks as a function of genome size and environmental challenge**. Boxplot of fitness reached at the end of adaptation. Network size, 19, 14, 9, 6, 4, is presented with a gray level, lighter gray representing larger network sizes. Larger Network size facilitates adaptation to higher fitness. To uncover the difference between highly adapted networks, the scale used is -log(1-Fitness).

**Figure 4:**
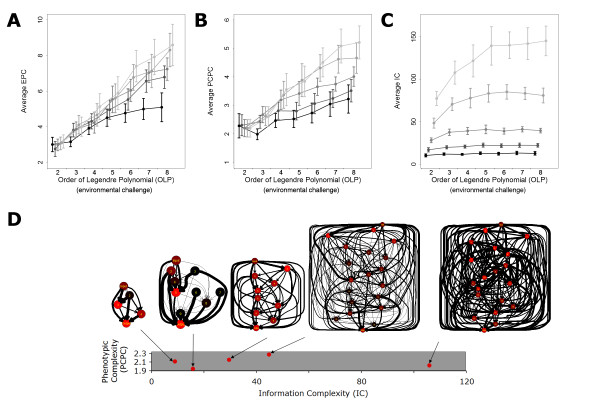
**Estimates of networks complexity as a function of both network size and environmental challenge**. A) Average Effective Phenotypic Complexity (EPC) estimated for different network sizes and environmental challenges (OLP). Network sizes of 4, 6, 9, 14 and 19 nodes are represented as decreasing shades of gray. Error bars are 95% confidence intervals. B) and C) same as A but for the metrics Principal Component Phenotypic Complexity (PCPC) and Informational Complexity (IC). D) Networks adapted to an OLP of 2 and having similar fitness and PCPC are presented with their respective value of PCPC and IC. On the networks graphed, the width of a connection reflects the impact of the underlying weight on fitness. A large width reflects a weight that impaired fitness largely when mutated. Large differences in the internal structuring of networks affected their IC but not their phenotypic complexity that remained more linked to the function performed.

**Figure 5:**
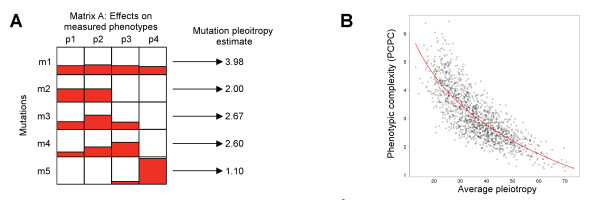
**Restricted pleiotropy and complexity**. A) Matrix illustrating pleiotropic effect of mutating weights of connections and nodes on network phenotype. The pleiotropic effect of a mutation was measured through the mean and variance of its effects on networks phenotypes. An average pleiotropy was computed for each connection and node weights in the network (generating matrix A) and averaged over all weights to compute the network pleiotropy. B) Correlation between the estimates of PCPC of phenotypic complexity and network pleiotropy for all networks. In red, a power law fit to the data.

**Figure 6:**
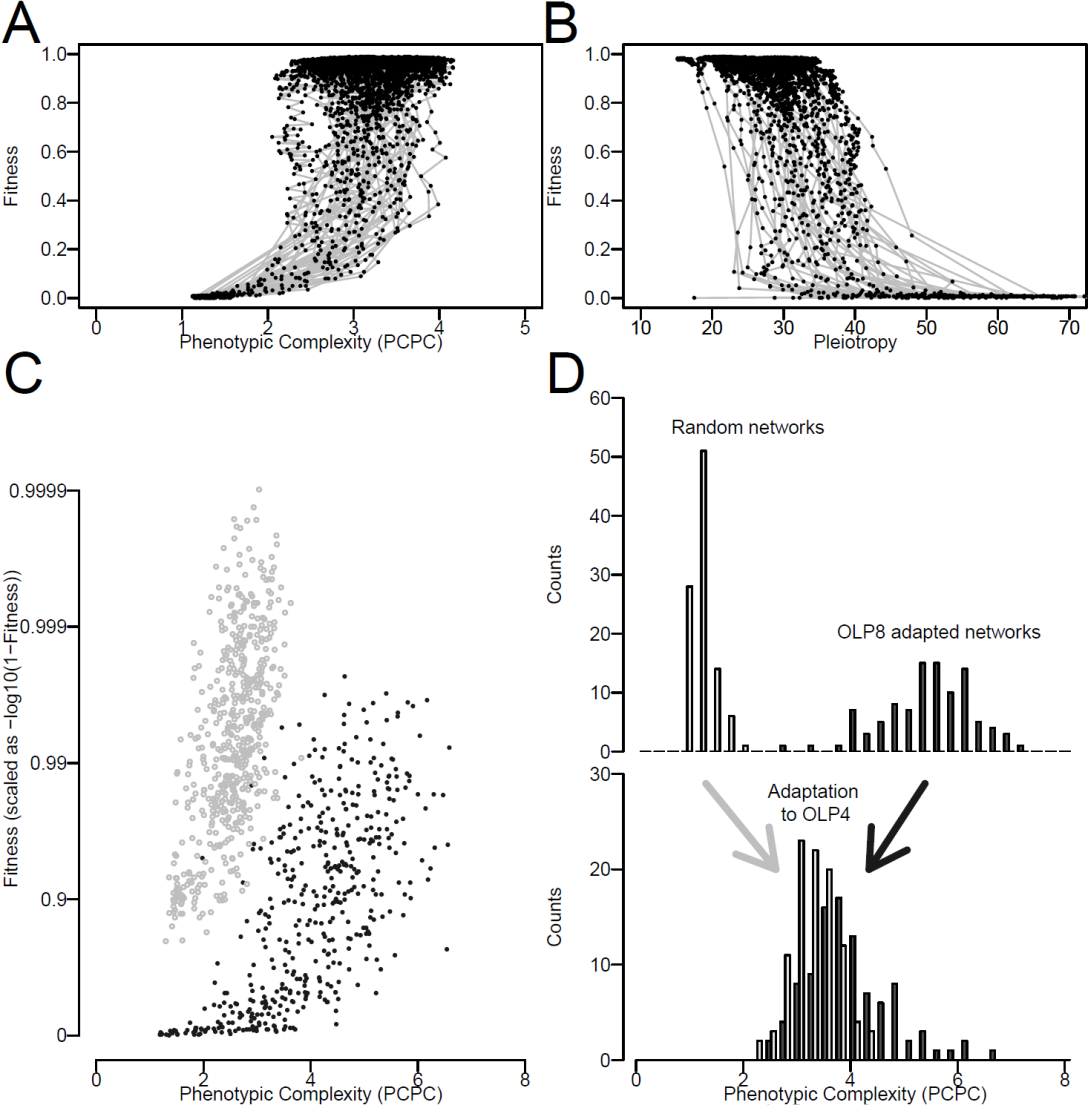
**Optimization of phenotypic complexity (PCPC)**. A) Evolution of fitness against PCPC for 50 19-nodes networks evolved on OLP 4 during the adaptive process. Gray line represents the fitness versus PCPC trajectory of the population and black dots the position of the most common genotype in the population each 10^4 ^generations. High PCPC was required to reach high fitness. B) Same as in A except that the average pleiotropy of networks was plotted instead of their PCPC. C) Final fitness reached under an "intense selection" adaptation process after 10^6 ^generations in two environments (OLP 3, light gray and OLP 8, dark gray) for networks of varying sizes as a function of evolved complexity PCPC. For each OLP a different range of optimal PCPC evolved and maximized fitness. D) Distribution of PCPC estimated on 100 random networks and 100 networks adapted to an OLP of 8 (top panel). Distribution of the PCPC estimated on networks, derived from the previous ones, after adaptation to OLP 4. PCPC converged, either up or down, towards an intermediate and optimal value.

**Figure 7:**
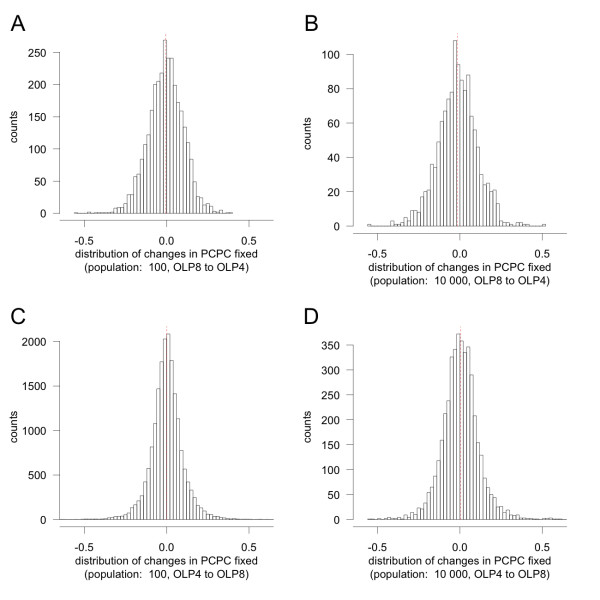
**Distribution of changes in complexity (PCPC) fixed during the adaptive process A) 12 populations of size 100 were adapted to an OLP of 4 in three independent replica from 4 networks previously adapted to an OLP of 8 for 10 million generations**. The populations were evolved under an "adaptive dynamics" process of adaptation (low mutation rate such that populations were always monomorphic unless a single mutant occurred and got either lost of fixed). The changes in PCPC of all the fixed mutations were then recorded. We focused on changes occurring while fitness of the network population changed from 10% to 60%, to avoid any effect due to the stabilization of the changes in PCPC in early and late phases of adaptation. The red dotted line represents the mean change in PCPC. B) Same as in A with a population size of 10 000. C) Same as in A on 5 networks (in 5 replicates each) adapting from an OLP of 4 to an OLP of 8 with a population of 100. Changes in PCPC were recorded while fitness changed from 5% to 30%. D) Same as in C with a population size of 10 000.

In the first dataset (Figure 3, 4, 5), we used our primary method to generate the evolutionary process. Networks evolved for 10 millions of generations or until they reached fitness of 0.99, with a population size of 500. EPC was then computed following the method described bellow. If in the process of computing the EPC a better fitness was found the best fitness network was stored and readapted for 10 million generation and computation of EPC started completely. In the second dataset (Figure 6), the "intense selection" evolutionary process was used for 10 millions of generations. The "adaptive dynamics" evolutionary process was used to follow the changes in complexity along the adaptive walk either starting from random networks or starting from networks previously adapted to an OLP of 8 and then shifted to an OLP of 4 (Figure 7).

### Network complexity

#### Principal component phenotypic complexity

For a given network, the different outputs of 1,000 mutations having more than 1% effect on fitness were recorded (using all mutations provided similar estimates, but due to the existence of fully neutral mutations resulted in a higher estimation variance and in the failure to estimate PCPC for some networks). For each mutation and for each of the P = 100 fitness linked phenotype (i = 1, 2,..., 100), the deviation between the mutant and wild type output N(g(i)) was recorded. A principal component analysis (PCA) was then performed in R, with a correlation approach. The eigen values, *λ_i_*, representing the variation explained by each of the P axes of the PCA were then used to estimate the number of effective dimensions, or PCPC. Because log(*λ_i_*) values tended to follow some uniform distribution, as in many system biology models [31], it was not easy to identify clear thresholds between axes contributing to the observed variance and those that did not. However, one could compute the number of effective dimension *n *or PCPC that would produce a variance among the contribution of all the P axes similar to the one observed (var(*λ_i_*)), assuming that *the n *first axes contribute equally to the observed variance and the remaining P-*n *have no contribution. Hence, as previously shown [11],

(6) $$PCPC=n=P\;∕\;\left(1+CV{\left(\lambda_{\;i}\right)}^{2}\right)$$

*CV*(*λ_i_*) being the coefficient of variation of *λ_i_*.

PCPC can be used to estimate the number of effective dimensions in Fisher's geometric model even when individuals sharing the same fitness are not equidistant from the optimal phenotypic combination (they define circular fitness isoclines in two dimensions) [11]. The resulting estimate of PCPC corresponds to the number of dimension of Fisher's geometric model with equidistant fitness isoclines that would have a distribution of mutation fitness effects similar to the one observed in the original phenotypic space in which fitness isoclines may be ellipsoidals [11].

#### Effective Phenotypic Complexity

In Fisher geometric model of adaptation, population evolves towards equilibrium fitness values defined by population size and the dimension of the phenotypic space or EPC. The best network at the end of adaptation was then used to initiate new populations of reduced size (6, 10, 30, 60 and 100), which were evolved for 10 millions of generation to be sure that equilibrium would be reached. Fitness was recorded over the last 5 million generations and the observed decay of fitness with population size was fitted by the theoretical prediction to estimate EPC [12] (Figure 4A).

(7) $$f\left(N\right)\propto {\left(1-\frac{1}{2N-1}\right)}^{EPC}$$

Indeed EPC is a composite index which corresponds to the ratio of dimensionality and an epistasis parameter named Q in reference [12]. For the sake of simplicity we assumed Q = 1 (we found evidence that Q < 2, but we could not find any evidence for a change in Q among networks (data not shown)). As we are not focusing on absolute values of EPC, this does not affect our results.

#### Informational Complexity

The quantity of information stored in a genome and transmitted to the next generation has been used as a measure of organismal complexity [10]. If more information is necessary to build up an organism, the organism is more complex. However, genome size alone may not be adequate because some parts may not encode any information. To account for the information content of a locus, its effects on fitness must be assessed. If all alleles at a locus had the same fitness, then there is no constraint on that locus: all alleles are equally probable and the information content is null. If all alleles but one are lethal then the information content is maximal, as the non-lethal allele will always be found at that locus. Because our loci are quantitative (the real value of a connection or node weight), we estimated the quantity of information (IC) by sampling the fitness effects of an observed weight. For each weight, we estimated the fitness for 600 interspersed values of the weight around its observed value (in a [-3, + 3] interval). To calculate the IC, we calculated the probability to observe each of these possible values in a population at equilibrium (because hitchhiking in adapting populations will affect the results [32]). Published measures of IC have used an infinite population size with mutation rate μ to get the equilibrium frequencies [32], but the resolution is not trivial. Hence rather than studying the equilibrium probability of each weight value in an infinite population size with a given mutation rate, we studied the probability to observe a given weight value in a finite population of size N with a very small mutation rate. The two approaches focus on different noise parameters in the transmission of information. The first one focuses on mutation rate, and the other one on genetic drift, but both have very similar behavior. The benefit of the drift approach is that it allows the exact computation of the of the frequency of an allele in an equilibrium population [30], as long as we know the fitness values associated with each allele.

For a given weight, j, the fraction of populations evolving with size N having the i^th ^value for that weight and the associated fitness f_j,i _is just:

(8) $$p\left(j,i\right)=\frac{{f_{j,i}}^{2N-2}}{\overset{600}{\underset{k=1}{\sum }}{f_{j,k}}^{2N-2}}$$

the entropy for that weight is then

(9) $$H\left(j\right)=-\overset{600}{\underset{k=1}{\sum }}p\left(j,i\right)\;Log_{600}\left(p\left(j,i\right)\right)$$

and the information content

(10) $$IC\left(j\right)=1-H\left(j\right)$$

The information content of a network was hence defined as the sum over all the w weights

(11) $$IC=\overset{w}{\underset{j=1}{\sum }}\left(1+\overset{600}{\underset{i=1}{\sum }}\left(\frac{{f_{j,i}}^{2N-2}}{\overset{600}{\underset{k=1}{\sum }}{f_{j,k}}^{2N-2}}Log_{600}\left(\frac{{f_{j,i}}^{2N-2}}{\overset{600}{\underset{k=1}{\sum }}{f_{j,k}}^{2N-2}}\right)\right)\right)$$

The larger the N, the higher the information content, as any slightly deleterious mutation is easily filtered by natural selection. To be able to uncover the differences among networks and to be able to compute the solutions we chose an N of 100.

### Network Pleiotropy

To estimate pleiotropy, we used a method similar to the one used to estimate PCPC. We sampled one hundred mutations per mutable entity, w_i_, of the network and averaged their effect, to obtain ${\bar{\mu }}_{i,p}$ (average squared deviation from the non-mutated network output) on each of the P = 100 network outputs or fitness-linked phenotype. We then estimated how many effective phenotypes were affected by a given weight, w_i _by studying the coefficient of variation of these ${\bar{\mu }}_{i,p}$.

(9a) $$Pleio\left(w_{i}\right)=P\;∕\;\left(1+CV{\left({\bar{\mu }}_{i,p}\right)}^{2}\right)$$

Similarly to PCPC, a pleiotropy of 3 meant that the mutation had a distribution of effects on traits equivalent to a mutation that would affect 3 traits identically and none of the other traits (Figure 5A). The network pleiotropy was then computed as the average pleiotropy across all mutable entities. As there are 100 fitness-linked phenotypes, our measure of pleiotropy ranges from 1 to 100.

### Network Modularity

To estimate modularity, we used the bipartite leading eigen vector approach [33] with an implementation kindly provided by J. Zhang. This method required a boolean matrix of connectivity in which nodes are either connected to a phenotype or not. Similarly to the analysis of pleiotropy, to generate such a matrix from our networks, for each weight an average effect on each phenotype was computed based on 1,000 mutations on that weight. A weight was connected to a phenotype if mutations shifted the phenotype value by at least 0.002 (several threshold were used and gave similar results). Such a shift generates a fitness loss that is perceivable by natural selection with our evolving population of 500. The value of modularity Q was retained for each network. However because absolute values of Q are also dependent on the connectivity of the matrix, similarly to Wang et al (2010) a normalized modularity was computed by comparing the observed Q with that of matrices in which the phenotype affected by each weight were randomly chosen, and normalizing by the variance of Q among the random matrices (to generate a Z-score).

## Results

Networks were evolved under an asexual mutation-selection-drift process and selected to match OLP's of the order of 2, 3, 4, 5, 6, 7 and 8. We examined network sizes of 4, 6, 9, 14 and 19 nodes. For each combination of network size and OLP, thirty to eighty populations were started from a random network and evolved until they either reached fitness of 0.99 or evolved for at least 10 million generations at a population size of 500. Populations of networks with an absolute fitness of less than a threshold of 15% were not retained, as they do not match properly the imposed challenge (other threshold values were used and provided similar results). With low OLP's, all networks evolved to high fitness values and matched accurately their reference function. With increasing OLP's, fewer networks reached a fitness greater than 15%, and those that did, especially the smaller ones, attained a lower fitness. For instance, at an OLP of 8, only 29% of the networks with 4 nodes exceeded 15% while 82% of the networks with 19 nodes did; of these, average fitness was 74% in the 4-node networks and 96% in the 19-node ones. Hence, increased network size facilitates adaptation to increased environmental challenge (Figure 3).

PCPC, EPC and IC were all found to correlate positively with the size of the network and the environmental demand (OLP) (Figures 4A, B, C). All three metrics increased close to linearly with OLP when networks were able to evolve high fitness. However, the similarity between the three metrics weakened with a more detailed comparison.

Despite the fact that they are measured with radically different methods, the two estimates of phenotypic complexity PCPC and EPC correlated strongly (r = 0.69). IC correlated to the other two metrics, but to a lesser degree (r = 0.50 with PCPC and r = 0.34 with EPC). Network size was able to explain 85%, 20% and 8% of the variance of IC, PCPC and EPC, while OLP explained 2%, 40%, and 57% of the variance, respectively (Figure 4). Moreover, the impact of network size on PCPC and EPC was partly driven by the inability of small networks to reach high fitness and high complexity in high OLP. Thus, while EPC/PCPC were much more influenced by the OLP, IC and its hybrid features was much more sensitive to bottom up properties of network such as network size.

Although network size did correlate with all three metrics of complexity, its inability to explain the variance in PCPC and EC revealed its limitations in influencing the evolution of complexity. This effect was further illustrated when networks were challenged with the simplest demand of OLP equal 2 (Figures 4A, B). Increasing network from 4 to 19, which corresponds to a 15-fold increase in the number of evolvable weights (see Methods), had not effect on EC and PCPC (p = 0.22) (Figure 4D). Consistent with earlier measurements, network size also explained up to 88% of the variance of IC in these conditions.

To elucidate how connections within a network evolved, we examined the effects of changing randomly the connection and node weights on each of the 100 traits used to estimate the network fitness (see Methods). A change in a weight is equivalent to a mutation and its effects were quantified as a pleiotropic coefficient, based on the coefficient of variation of the magnitude change at each trait (Figure 5A). A mutation affecting equivalently all traits is universally pleiotropic with a coefficient of 100. A smaller coefficient indicated a mutation with restricted pleiotropy. The average pleiotropy of networks correlated negatively and strongly with metrics of complexity (Figure 5B) and restricted pleiotropy was associated with increased complexity. Thus, mutations in the simplest networks were maximally pleiotropic and affected most traits, while the ones in complex networks exhibited restricted pleiotropy by affecting differing subsets of traits. This negative correlation was highly significant across network sizes or OLP. The correlation was maximal for PCPC (overall correlation of -0.75 with a power law) but was still strong for EPC (r = -0.57) and IC (r = -0.56). Indeed such a link between phenotypic complexity and pleiotropy is fully compatible with the principal component analysis vision of complexity. For many independent axes to emerge, mutations must affect differentially the different traits, such that no absolute correlation exists among them.

The negative correlation between pleiotropy and our three metrics of complexity indicated that complexity evolved by incorporating mutations with restricted effects on the response of the networks. To determine whether the restriction also created structuring in which particular group of weights interacted preferentially with different subsets of phenotypes, we searched for modularity by applying the bipartite leading eigen vector approach to the matrix of connections from weight to phenotypes [33]. In this matrix, a weight was connected to a phenotype only if the average effect of mutations on that weight affected the phenotype value by 0.002 units, which corresponds approximately to an effect on fitness detectable by natural selection with the population size of 500. The modularity statistic Q correlated negatively with pleiotropy (r = -0.44) and positively with PCPC (r = 0.28), but the correlations were not very strong. This may be due to the dependency of Q to the density of the matrix used to compute it. We therefore computed a modified value of Q, which corresponds to the normalized difference between the observed value of Q and that of random matrices sharing the same connectivity (see Methods) or Z-scores. The presence of modularity was pervasive: 1296 out of 1368 networks had a higher than expected modularity at the 5% level. So restricted pleiotropy emerged partly due to the emergence of modularity. However, some further analysis of modularity, pleiotropy and complexity will have to be performed with greater care, as the statistical properties of these Z-score and of the basal level of the Q statistic remain to be uncovered.

Previous theoretical studies, analyzing the consequences of phenotypic complexity on evolution, have indicated that high complexity generates some costs such as an increased drift load [12,16,20,23-25]. As drift load is also the basis of our metric EPC, the evolution of higher EPC with increasing environmental demands (Figure 4) reveals directly the existence of some cost. Because complexity emerged in our networks despite the presence of a drift load, some associated benefits must have outweighed its cost. To unravel these benefits, we monitored the emergence of phenotypic complexity to determine if it conferred better adaptation and higher fitness. First, we analyzed the networks reported in Figure 6A and 6 found that complexity measured by PCPC both increased during the early phases of adaptation and correlated positively with fitness. Similarly, the level of pleiotropy decreased during adaptation (Figure 6B). Second, we generated and analyzed another dataset in which the selective pressure was kept intense such that very high fitness could be reached independently of the evolving population size (see Methods). The final fitness reached in this "intense selection" dataset again correlated positively with PCPC for each of the environmental challenges (Figure 6C) and the PCPC values reached were similar to the one observed previously. Third, we examined an additional dataset in which complexity emerged while being forced to remain below a threshold. Mutations producing networks with a PCPC higher than the threshold value were rejected. Once again the maximal fitness reached was very strongly positively correlated with the maximal PCPC allowed (data not shown). Those three approaches revealed that low complexity organisms are unable to reach very high fitness while organisms above a given complexity manage to (Figure 6A, C). For instance, in Figure 6A a network could reach a fitness higher than 40% only if its PCPC was higher than 2.2 and its pleiotropy lower than 45. Thus, when facing a demanding environment, networks could achieve a high fitness only if restricted pleiotropy emerged through the decoupling of mutation effects and allowed fine-tuning of its outputs.

However, an ever-increasing complexity, which could in theory lead to an ever-increasing fitness, was not observed (Figure 6C). This suggests that the cost of increasing complexity may have been substantial. While the emergence of high complexity was observed for networks adapted to high OLP, such high levels were never observed for lower OLP despite the permanent selective pressure we maintained in our "intense selection" dataset (Figure 6C). This outcome suggests that the failure to evolve high complexity result from the cost of high complexity and not from (i) a weakening selective pressure as fitness increases, or (ii) a mutational pressure due to an increased probability to decrease complexity by mutation. Indeed, both alternatives would generate a dependency of the complexity reached with the intensity of selection and/or the population size, but neither were observed (data not shown). Moreover, we looked at the distribution of changes of PCPC caused by random mutations and did not found any consistent pattern: networks had sometimes an excess of mutations increasing PCPC, sometimes a lack. This further discredits the role of biased mutational pressure in setting the complexity equilibriums. Finally, to test for the presence of a cost of complexity, we evolved networks to very high levels of complexity by selection in a very demanding environment (OLP = 8) and then switched them to a lower OLP (equal to 4). In all cases, we observed a gradual decrease in PCPC towards the average value reached by random network adapted to OLP = 4 (Figure 6D). Hence an optimal level of complexity was favored and resulted most likely from a balance between the benefits and the costs of complexity.

## Discussion

By evolving adaptive networks with different physical properties under different environmental conditions, we have been able to identify the determinants controlling the evolution of complexity. The three different measures of complexity we used correlated positively with one another, yet our analysis reveals that they captured different facets of complexity. We found that while IC captured the physical architecture used to accomplish a given task, PCPC and EPC were most useful in describing the integrated task the organism performs.

The measure of informational complexity or IC, being still connected to the physical constituents of the networks, was found to be mostly driven by network size and not to correlate well with the environmental challenge. For large networks, IC responded more to the environmental challenge (Figure 4C), presumably because inactivating unnecessary parts of the network was an achievable solution for large networks facing simple challenges. However, as soon as the intensity of the challenge increased the whole network was recruited and IC saturated. Overall, for all environments, the size of the networks was the principal determinant of IC. The latter outcome demonstrates dramatically that the same ecological problem can be solved by a variety of genetic solutions in these networks and raises the possibility that the more physical aspects of complexity may be more subject to the vagarities of historical contingencies [34]. This may be the reason why bottom up approaches of complexity do not match adequately our intuitive perception of complexity which is based on the observation of organisms phenotypes. For instance, the recent analysis of the genomes of *C. reinhardtii *and *V. carteri *[6] supports well the qualitative outcome of our study. While *C. reinhardtii *is a single-celled algae, *V. carteri *is multicellular, the two have similar genome size and number of genes. Thus, two organisms straddling the boundary of a major biological transition are able to manifest marked differences in phenotypic complexity while evolving minimal changes in their informational content.

The estimates of phenotypic complexity, PCPC and EPC strongly correlated with each other, which is remarkable because it indicates that mathematical derivations from an idealized model of adaptation such as Fisher's quantifies complexity in a manner similar to a statistical model of principal components. Although this may seem intuitive as both estimates are supposed to measure phenotypic complexity as defined in Fisher's geometric model, nothing suggested initially that such a model should apply to our networks. The high correlation we observed relies on the robustness of the estimators of complexity derived from Fisher's geometric model that we used. Other measures of dimensionality based on the distribution of mutation effects in Fisher's geometric model have been proposed [11], but they are dependent on assumptions of made on how mutations affect phenotypes [18,35] and have failed to be informative in our system (data not shown). Contrary to these estimators, EPC is independent of the mutation process [12] and is therefore more robust. PCPC which uses mutations to sample the phenotypic space directly, is also more informative in our case, simply because all phenotypes influencing fitness are known and the effect of thousands of mutations on all these phenotypes can be measured in artificial systems. As a consequence, PCPC relies on the full information of mutational effects on phenotypes and not only on summary statistics such as the mean and variance of mutation effects on fitness that are informative on the dimensionality of the space in Fisher's geometrical model only under restrictive conditions. (The correlation between PCPC and EPC suggests therefore that while Fisher's Model provides a relevant description of the behavior of the networks, the restrictive conditions required for the distribution of mutational effect on fitness to be informative on dimensionality are not met in these networks.)

Both PCPC and EPC responded consistently to the demands of the environment. Because the environment is perceived by the networks through the fitness consequences of natural selection, our results shift the evolutionary focus of complexity from bottom up or physical measures of complexity (genome size, number of cell types, network size) to top down or more ecological ones in which complexity is linked to the ecological niche. Physical measures of complexity alone are not adequate to capture complexity of the task performed because their mapping to phenotype was modulated as we found by pleiotropy.

Pleiotropy has been defined a century ago [36], yet, some large scale estimates of pleiotropy within an organism have only been provided recently [37]. Consistently with the results of our toy model, analysis of mouse QTL [38], or yeast, nematode and mouse knock outs [39] have suggested that pleiotropy was very restricted [37]. Mutations affected a fraction of the phenotypes measured and not all as considered classically for a long time in Fisher's geometric model. Moreover, similarly to what we found, some extensive modularity may contribute to the emergence of restricted pleiotropy [39]. While our results are compatible with these observations, they extend them by suggesting a more intimate connection between pleiotropy and phenotypic complexity.

The link between restricted pleiotropy and the emergence of phenotypic complexity provides both a mechanistic interpretation of complexity and a selective hypothesis underlying its evolution. Previous analyses of phenotypic complexity have mostly focused on the consequences of complexity on evolution, rather than on the selective forces acting on it. As such it appeared costly to have a high complexity due to a limited number of beneficial mutations [11,13,15,21], a limited rate of adaptation [7,11,21,22], or a higher drift load [12,16,20,23-25]. However, these models assumed that all organisms independently of their complexities could potentially reach the same maximal fitness. Here, we suggest that if strong correlations between mutations effects exist within an organism, this organism has low complexity because it can only explore a fraction of the phenotypic space [11]. Hence, it could not reach a high fitness when placed in a challenging environment. The way to reach a higher fitness is then to decouple mutation effects such that mutations affect a subset of phenotypes and not all. As a result the accessible phenotypic space becomes larger and its number of dimensions, *i.e*. the phenotypic complexity of the organism is increased.

Phenotypic complexity and restricted pleiotropy appeared to be under stabilizing selection due to a balance between their benefits and costs. Increasing complexity allows the organisms to wonder in a larger phenotypic space and closer to the optimal combination of phenotypes, but it also leads to a higher drift load [12]. The effects of drift limit the ability of the population to stay close to the optimal combination because it is harder to optimize more independent traits simultaneously. Both the costs and the benefits of complexity appear to be indirect (second order selection [40]) and weak: (i) if selection for complexity was direct, there would be a correlation between the effect of mutations on fitness and the ones on complexity, but no such correlation was found (data not shown), (ii) if selection for complexity was strong, changes in complexity fixed in the adaptive process should be mostly positive when selection favored an overall increase in complexity and negative in the opposite case. Yet, in both cases, the distribution of changes fixed was only slightly off-centered (Figure 7), with almost as many changes towards increased complexity than towards decreased complexity. Interestingly, in a Fisher's geometric model with fixed complexity, restricted pleiotropy was found to alleviate the costs of complexity in terms of adaptation rate [39]. Yet in the model developed here, we suggest that restricted pleiotropy emerges to expand the range of accessible combinations of phenotypes. Hence the benefit of restricted pleiotropy we monitored is linked to a change of the maximum fitness reached (despite a higher drift load) and presumably not to a change of the rate of adaptation. Whether networks with higher complexity tend to adapt faster or slower in our system and whether this effect could promote the evolution of complexity remain to be tested.

Finally, restrictive pleiotropy's link to complexity is consistent with Ohno's hypothesis for the evolution of complexity via gene duplication [41]. Much as how restrictive pleiotropy can decouple two phenotypes in our model, gene duplication allows the two gene copies to evolve freely. Sub-functionalization following a gene duplication (in which the two derived copies of a gene are required to replace the function of the ancestral gene) provides a perfect example of a mechanism by which pleiotropy can be reduced. Moreover, as the selection acting on complexity appears to be indirect in our system (Figure 7), our results are compatible with the idea that chance or genetic drift may play a role in the emergence of complexity [42]: some slightly deleterious changes in complexity may be required to facilitate the acquisition of subsequent beneficial mutations. This is equivalent to the idea that sub-functionalization may initially result from non-selective forces, but may in the longer term be recruited by natural selection to fine tune the adaptation and therefore support long term incremental evolution of complexity. Such a perspective will be subject to further studies.

## Conclusions

Using a model of adaptive neural networks, we have shown that phenotypic complexity evolved as a function of the demands of the selective pressures, rather than the physical properties of the network architecture, such as functional size. The phenotypic complexity we observed resulted from a selective balance between the costs associated with the optimization of many independent traits and the benefit provided by the exploration of a larger phenotypic space. Our model suggests hence both a selective process for the emergence of phenotypic complexity and a mechanistic model allowing its evolution: the emergence of restricted pleiotropy. Our results therefore show that complexity may be more predictable, and understandable, if analyzed from the perspective of the integrated task the organism performs, rather than the physical architecture used to accomplish such tasks. Thus, top down metrics emphasizing selection may be better for describing biological complexity than bottom up ones representing size and other physical attributes.

## Authors' contributions

HLN and OT conceived and design the experiments. HLN performed the experiments. HLN, LC and OT wrote the paper.
